# Research progress of biomarkers in evaluating the severity and prognostic value of severe pneumonia in children

**DOI:** 10.3389/fped.2024.1417644

**Published:** 2024-10-01

**Authors:** Weihua Gong, Kaijie Gao, Zhiming Shan, Liu Yang, Panpan Fang, Ci Li, Junmei Yang, Jiajia Ni

**Affiliations:** ^1^ Department of Clinical Laboratory, Children's Hospital Affiliated to Zhengzhou University, Zhengzhou Key Laboratory of Children's Infection and Immunity, Zhengzhou, Henan, China; ^2^ Department of Detection and Diagnosis Technology Research, Guangzhou National Laboratory, Guangzhou, China

**Keywords:** severe pneumonia, child, biomarkers, prognosis, severity

## Abstract

Pneumonia is a serious and common infectious disease in children. If not treated in time, it may develop into severe pneumonia. Severe pneumonia in children is mainly characterized by hypoxia and acidosis, often accompanied by various complications such as sepsis and multiple organ dysfunction. Severe pneumonia has a rapid onset and progression, and a high mortality rate. Biomarkers assist clinicians in the early diagnosis and treatment of patients by quickly and accurately identifying their conditions and prognostic risks. In this study, common clinical and novel biomarkers of severe pneumonia in children were reviewed, and the application value of biomarkers related to the severity and prognosis of severe pneumonia in children was evaluated to provide help for early identification and precise intervention by clinicians.

## Introduction

Pediatric pneumonia is a common infectious disease in the clinic. Infants and young children are susceptible to severe pneumonia with high mortality rates. In 2019, 740,180 children under the age of five died from pneumonia worldwide, accounting for 14% of all deaths among children under five years of age (1). Data shows that the majority of childhood pneumonia deaths are caused by severe pneumonia (2). Due to their unique anatomical characteristics, children have low anti-infection ability and are prone to respiratory infections. Once infection occurs, it can easily spread to the lungs and develop into pneumonia or severe pneumonia. Data from the World Health Organization (WHO) and previous researches indicate that pneumonia is the primary infectious cause of death in children globally. Children and families worldwide struggle to protect themselves from pneumonia, a challenge particularly evident in developing countries (3). With rapid onset and progression, and high fatality rate, severe pneumonia in children can easily induce serious complications, such as heart failure and sepsis, leading to multiple organ dysfunction and even life-threatening complications. The continuous release of inflammatory mediators in the body of patients with pneumonia causes inflammatory storms, coupled with the inherent weakening of immune function and T cell apoptosis, resulting in immune suppression, immune dysfunction, and an increased risk of poor prognosis (4, 5). The severity of pneumonia is closely related to the immune function and inflammatory response of the body. Dynamic monitoring of immune and inflammatory biomarkers plays a crucial role in assessing the severity and prognosis of severe pneumonia in children.

The occurrence of severe pneumonia is related to various factors such as pathogen infection, underlying diseases, weakened immunity, and environmental pollution. *Streptococcus pneumoniae* is a common cause of bacterial pneumonia in children and a leading cause of death from respiratory infections in children under five years of age. Respiratory syncytial virus (RSV) infection is the most common cause of viral pneumonia. Severe pneumonia can be caused by a variety of pathogens, of which bacterial pneumonia is the most common. In recent years, owing to changes in the climate and environment, pathogens associated with severe pneumonia in children have also changed. Studies have shown that the number of children infected with mixed or co-infected pathogens is increasing (6, 7). Early prediction of the occurrence of severe pneumonia and timely prognosis has become the focus of clinicians.

With the development of medical and health technologies, significant progress has been made in the diagnosis and treatment of severe pneumonia in children. The mortality rate of pneumonia decreased from 4 million in 1981 to over 1 million in 2013, but still accounts for nearly one-fifth of global child deaths (8–10), which is associated with the inability to accurately assess the disease. We usually diagnose pneumonia patients by comprehensively evaluating symptoms, vital signs, laboratory examinations, and radiological examinations. Typical signs and symptoms include shortness of breath, cough, fever, anorexia, and dyspnea (11, 12). However, symptoms and vital signs are not specific for the diagnosis of pneumonia, and there are still some patients who do not have typical symptoms and signs (13). The use of laboratory examination indicators could distinguish the types of pneumonia infections, such as bacterial pneumonia, viral pneumonia, and non-infectious respiratory diseases. Radiological examination indicates the presence of pulmonary infiltrative shadows on chest radiography, as well as any possible symptoms such as fever, cough, sputum, chest pain, and dyspnea, which can be diagnosed as pneumonia (14, 15). WHO has revised the classification of childhood pneumonia. For children under 5 years old who have cough and/or difficulty breathing, with or without fever, pneumonia is diagnosed by the presence of either fast breathing or chest wall depression (3, 16). When a child with pneumonia shows any general signs of danger, such as inability to feed or drink, loss of consciousness, hypothermia, and convulsions, a diagnosis of severe pneumonia can be made (17, 18). However, there is currently no unified diagnostic standard for severe pneumonia in children, both domestically and internationally, which makes it difficult to accurately predict the severity of the disease. Some patients have atypical clinical manifestations, which increases doctors’ recognition. If a patient's condition is not promptly evaluated, it is easy to miss the optimal treatment time, which affects the patient's prognosis. Therefore, it is necessary to identify biomarkers that can accurately determine the condition and prognosis of children with pneumonia.

Biomarkers have certain reference values for the early assessment of disease severity and prognosis, facilitating doctors to timely evaluate the treatment effect of patients. Biomarkers provide objective information about the host's involvement in immune responses, replacing the limitations of atypical symptoms and signs, to some extent, bringing great benefits to doctors and patients. In recent years, many studies have explored biomarkers to assess the severity and prognosis of severe pneumonia in children (19–21). It should be pointed out that a single biomarker often cannot simultaneously balance sensitivity and specificity. Currently, no biomarker is the best indicator to evaluate this condition. In the era of precision medicine, monitoring biomarkers and predicting the body's response to drugs, in order to choose the better treatment. Strengthening the evaluation of the severity and prognosis of severe pneumonia in children and early intervention in high-risk children is of great help in halting disease progression, preventing complications, and reducing mortality. Therefore, we reviewed common clinical and novel biomarkers of severe pneumonia in children (Table 1), helping clinicians accurately identify children with severe pneumonia at an early stage, intervene as soon as possible, simplify the diagnosis and treatment process, and improve patient prognosis.

**Table 1:** Biomarkers of severe pneumonia.

Common clinical biomarkers	Novel biomarkers
CRP	ProADM
PCT	Copeptin
NLR	suPAR
PLR	TREM-1
RDW	PSP
ILs	
SAA	
DD	
PA	
LDH	

CRP, C-reactive protein; PCT, Procalcitonin; NLR, Neutrophil-lymphocyte ratio; PLR, Platelet lymphocyte ratio; RDW, Red blood cell distribution width; ILs, Interleukins; SAA, Serum amyloid A; DD, D-dimer; PA, Prealbumin; LDH, Lactate dehydrogenase; ProADM, Pro-adrenomedullin; suPAR, Soluble Urokinase Plasminogen Activator Receptor; TREM-1, Triggering receptor expressed on myeloid cells 1; PSP, Presepsin.

## Common clinical biomarkers

### C-reactive protein (CRP)

CRP is an acute phase-responsive protein produced by liver cells under the stimulation of pro-inflammatory factors. CRP acts by activating the immune system and stimulating phagocytes, which is a common biomarker of acute infection. The CRP content in the serum of healthy individuals is very low, generally less than 10 mg/L. After being stimulated by tissue damage, infection, or inflammation for 4–6 h, plasma CRP level increases, reaching a peak at 48–72 h, which may continue until the 7th day (22). After 3 days of inflammation disappearance, CRP decreased to normal levels. The half-life of CRP is 18–19 h. Therefore, plasma CRP levels are influenced by the body's production time and rate, rather than the elimination rate.

Currently, CRP level is a common indicator for the diagnosis of bacterial infections, efficacy evaluation, and disease assessment. Two prospective longitudinal cohort studies found that CRP levels were lower in children with pneumonia three days before symptoms appeared, suggesting that CRP testing after three days was more meaningful in assessing disease severity (23). Among patients diagnosed within the first 48 h, the inflammatory response in the body is in an increased phase, and CRP levels may still be low. Three or more days after the onset of symptoms, the levels of some biomarkers in the patient's body (such as PCT, IL-6, and IL-8) may have decreased, while CRP levels may peak and persist for a while (20). Therefore, the increase and decrease in CRP levels often lag behind other biomarkers. In patients with symptoms lasting for three days or longer, using CRP to evaluate disease severity and prognosis is a better choice.

### Procalcitonin (PCT)

PCT, a glycoprotein encoded by the CALC-I gene on chromosome 11, is a procalcitonin precursor secreted by thyroid C cells. It can be expressed in the neuroendocrine cells of the thyroid, lung, and pancreas of normal individuals with extremely low levels, generally less than or equal to 0.1 ng/ml (24). PCT was first identified as a biomarker for bacterial infections in 1993, with strong specificity for the early diagnosis and disease assessment of bacterial infections (19, 25). PCT increases within 2–3 h of infection and peaks at 12–24 h. It increases in typical bacterial infections, whereas increases in viral or atypical bacterial infections are not significant or do not increase (19). The half-life of PCT is approximately 20–24 h, and the dynamic monitoring of changes in PCT concentration can better evaluate the degree of disease development. The characteristics of CRP and PCT are listed in Table 2.

**Table 2:** Characteristics of CRP and PCT.

Biomarker	Normal value	Production organs	Half-life (h)	Production time from infection (h)	Peak time from infection (h)
CRP	<10 mg/L	Liver	18–19	4–6	48–72
PCT	<0.1 ng/mL	Lung, liver, kidney, intestine, muscle, adipocyte	20–24	2–3	12–24

CRP, C-reactive protein; PCT, procalcitonin.

Research has shown that PCT is the best diagnostic biomarker for *S. pneumoniae* pneumonia in children. Plasma PCT greater than or equal to 0.25 ug/L often indicates bacterial infection, and the possibility of viral infection and co-infection cannot be ruled out. The more severe the infection, the higher the PCT elevation. A prospective cohort study found that plasma PCT greater than 2 ug/L can serve as a biomarker for severe pneumonia in children (26). It plays an important role in the early assessment of patients with severe pneumonia and in predicting their prognosis. Some studies have also found that one-week dynamic monitoring of PCT in patients with severe pneumonia accurately predicts the direction of disease development (27, 28).

### Blood routine-related indicators

Routine blood tests are one of the most common clinical tests, in which white blood cells (WBCs), neutrophils (NEUTs), lymphocytes (LYMPHs), and platelet-related indicators are often used as markers of infection and inflammation. Abnormal blood routine-related indicators often indicate that some diseases, especially in the diagnosis and treatment of infectious diseases, are of great significance.

### Neutrophil-lymphocyte ratio (NLR)

NLR integrates two subtypes of WBCs and is a common indicator for evaluating systemic inflammatory response. NEUTs are the first to drive immune responses after pathogen infection (29). Simultaneously, NEUTs recruit other immune cells and exert immune effects by secreting cytokines and chemokines. Excessive proliferation and activation of NEUTs induce lymphocyte redistribution and accelerate apoptosis and marginalization, leading to a decrease in anti-infection ability. LYMPHs reflect the stress state of stress in the body. The stronger the stress response, the lower is the lymphocyte level (30, 31). NLR assesses inflammatory status and immune response intensity, with a normal range of 1–2. An increase in NLR often indicates a heightened inflammatory response and weakened immune response.

Increased NLR was found to be an independent risk factor for 30-day mortality in patients with pneumonia (32). It has a promising application value in predicting the severity of pneumonia and sepsis. Treatment with NLR greater than 6.11 has been reported to improve the prognosis of patients with severe pneumonia (33). The prognosis of patients is poor when NLR is greater than 28.3 (34). If the NLR continues to rise or does not improve, it suggests that the treatment is not effective and is a sign of poor prognosis. Therefore, NLR is a reliable predictor of the severity and prognosis of severe pneumonia in children.

### Platelet lymphocyte ratio (PLR)

Platelet is a non-nucleated cell produced by megakaryocytes and is mainly involved in coagulation and hemostasis processes. With the continuous advancement of medical and health technology and people's cognitive levels, an increasing number of studies have focused on the role of platelet in infection and immunity. Platelet plays a sentinel role in infectious diseases and a cohesive role in the immune response, inflammatory response, and anticoagulation process (35). When the body is infected, platelet migrates to the site of infection, preventing the spread of pathogens and the invasion of extravascular bacteria. After platelet activation, the body releases large amounts of inflammatory mediators, inducing the activation of immune cells, such as NEUTs, monocytes, and macrophages, and participates in the inflammatory response.

The PLR integrates lymphocyte and platelet parameters, reflecting the degree of platelet activation. The degree of PLR increase is positively correlated with the degree of platelet activation and risk of thrombosis. PLR is increased in inflammatory reactions, which might be considered as a potential biomarker of infectious diseases. High PLR levels predict the occurrence of severe pneumonia (36). Infection with severe pneumonia, alveolar ventilation, and arousal dysfunction, results in decreased blood oxygen content, and increased secondary platelet count. The number of platelets decreases in the later stages of infection as the disease progresses (37). The PLR was monitored to assess the severity of severe pneumonia in children. However, there are few studies on PLR and severe pneumonia in children; therefore, whether PLR accurately predicts the severity and prognostic value of severe pneumonia in children needs to be further verified.

### Red blood cell distribution width (RDW)

RDW is an indicator that reflects the heterogeneity of red blood cell size and is commonly used in the diagnosis of anemia. Recently, RDW exhibits high diagnostic efficacy and prognostic value in sepsis, heart disease, and cancer, especially in predicting the prognosis of infectious diseases (38, 39). The body may affect the increase in RDW through various mechanisms, such as oxidative stress and inflammatory responses. It has also been shown to be an independent predictor of mortality in critically ill individuals (40). RDW is associated with the prognosis of severe pneumonia in children, which usually indicates a poor prognosis (41). RDW may provide a reference for assessing the severity and prognosis of severe pneumonia in children, but further research is needed to determine within what reference range RDW can accurately assess the adverse prognosis of patients with severe pneumonia.

### Interleukins (ILs)

ILs participate in the interaction between WBCs and immune cells by activating or regulating immune cells. IL-6 is a representative pro-inflammatory cytokine, secreted by LYMPHs or monocyte macrophages and belongs to the acute phase response protein. IL-6 is also an endogenous heat source that is associated with persistent high fever in patients. The serum level of IL-6 in healthy individuals is extremely low, and the level increases when the host is infected. The IL-6 levels increase significantly in severe infection, and its continuous rise often indicates a poor prognosis. IL-6 levels increased in response to various inflammatory responses, such as rheumatoid arthritis and multiple myeloma; therefore, its diagnosis is less specific (42, 43). When clinical symptoms disappear, IL-6 level rapidly decreases and its half-life is short. IL-6 is an important predictor for evaluating the severity of severe pneumonia and treatment failure and has been widely studied in clinical practice (44, 45). IL-10 is a representative anti-inflammatory cytokine. Studies have reported that an IL-6 to IL-10 ratio greater than five predicts the occurrence of severe pneumonia in children (46).

### Serum amyloid A (SAA)

SAA is a non-specific acute reaction protein synthesized by the liver. Serum SAA level is extremely low (<10 mg/L) in healthy individuals. The level of SAA significantly increases during the acute phase of infection, and the magnitude of the increase is related to the severity. SAA also plays an important role in the early diagnosis of viral infections, and its increase is most obvious after viral infection (47, 48). SAA has strong timeliness, and it can be detected up to 1,000 times in the early stages of infection (5–6 h). The half-life of SAA is relatively short (approximately 50 min) and decreases rapidly during the recovery period from infection. The serum SAA level in children with pneumonia increases, and the degree of this increase is negatively correlated with the progression and prognosis of children (49).

### D-dimer (DD)

DD is the smallest fragment produced by fibrin degradation and reflects the severity of infection and early coagulation function. After infection, on the one hand, it promotes the release of various inflammatory cytokines, accelerates the damage of the vascular wall, and activates the coagulation system. However, bacterial infections accelerate fibrin degradation. Increased DD indicates activation of the coagulation system and secondary fibrinolysis, which are biomarkers of hypercoagulability and fibrinolysis in the body. An increase in plasma DD levels is associated with disseminated intravascular coagulation (DIC), thrombosis, liver disease, and severe sepsis (50). The level of DD in the plasma of patients with severe pneumonia significantly increases, and the degree of increase is strongly correlated with poor prognosis and is not affected by factors such as age, sex, or region (51, 52). Children with high levels of DD are more likely to develop severe illness; therefore, clinicians closely monitor their condition by monitoring DD levels to reduce the possibility of progression.

### Prealbumin (PA)

PA is a glycoprotein synthesized in the liver and has a short half-life of approximately 12 h. It plays an important role in the occurrence and development of inflammation and repair of damage in the body, and is a key molecule that activates the immune system. Inflammation, infection, trauma, and malnutrition cause a decrease in serum PA concentrations. During an acute infection, serum PA eliminates harmful substances produced by the body, leading to a rapid decrease in serum PA levels. The expression of serum PA rapidly decreases during acute infection and the degree of decline is positively correlated with the degree of infection. In severe infection, the synthesis and absorption of nutrients are slowed down, which also causes a decrease in serum PA levels. Some data have shown that PA is a good marker for judging the severity and prognostic value of COVID-19 (53). Research has shown that serum PA concentration in children with severe pneumonia infection decreases (54, 55). There is a correlation between the decrease in serum PA levels and concurrent myocardial damage in children with severe pneumonia. Serum PA levels reflect the condition of the children and indicate their prognosis.

### Lactate dehydrogenase (LDH)

LDH is a cytoplasmic enzyme produced by anaerobic respiration. Traditional research has suggested a close correlation between LDH levels and myocardial injury. With the continuous deepening of research, it has been found that serum LDH levels are elevated in many inflammatory reactions, such as hematological diseases, heart failure, and respiratory diseases. Recently, the relationship between LDH levels and pneumonia has gradually been emphasized (56, 57). When severe pneumonia occurs, the body is in a state of hypoxia, which promotes the accumulation of acidic metabolites and increases LDH concentration. In contrast, the lung tissue of children with severe pneumonia is damaged, causing an increase in serum LDH levels. Previous studies have reported that LDH levels above 258.46 U/L have a higher sensitivity (76.9%) and specificity (62.1%) in predicting severe pneumonia (56). High LDH levels are a risk factor for death in children with severe pneumonia.

## Novel biomarkers

### Pro-adrenomedullin (ProADM)

ProADM, originating from the middle segment of vasoactive peptide precursor in adrenal pheochromocytoma, is a precursor of adrenomedullin (ADM). ADM has a short half-life and is difficult to detect owing to its binding protein. ProADM has a stable structure and is commonly used to represent the level of ADM. It is expressed in various tissues and organs and has vasodilator, anti-inflammatory, and antibacterial activities. Hypoxia and microbial stimulation lead to increased expression of ProADM, a common biomarker of respiratory infection (58).

Compared with traditional biomarkers, ProADM has a promising predictive role in the assessment of severe outcomes in adult respiratory infections (59). Scholars have found that compared with CRP and PCT, ProADM is more valuable in evaluating the severity of pneumonia in children, which is attributed to ProADM not relying on different types of pathogic infection (60, 61). ProADM is an important predictor of prognostic risk in children with severe pneumonia, and serum ProADM levels are negatively correlated with patient severity and prognosis (62). ProADM is more capable of predicting disease severity and is a novel biomarker worth studying. The predictive value of novel biomarkers for patients with severe pneumonia is shown in Table 3.

**Table 3:** Predictive value of novel biomarkers for severe pneumonia in patients.

Biomarker	Patients	Comment	Ref.
ProADM	Adults	ProADM has a promising predictive effect on the outcome of respiratory infections.	(59)
ProADM	Children	ProADM is associated with the severity of pneumonia in children.	(60)
ProADM	Children	ProaADM seems a reliable and available predictor for pneumonia.	(61)
ProADM	Children	Serum ProADM level is negatively correlated with the severity and prognosis of pneumonia in children.	(62)
Copeptin	Adults	Copeptin is associated with adverse clinical course in patients with pneumonia.	(63)
Copeptin	Adults	The level of Copeptin increases with the severity of respiratory infections, especially in pneumonia patients.	(64)
Copeptin	Children	Copeptin appears to be a reliable and usable predictor for assessing the severity and prognosis of childhood pneumonia.	(65)
suPAR	Children	SuPAR can be used as a predictive indicator for the severity of pneumonia in children.	(66)
suPAR	Children	An increase in suPAR is observed in pneumonia, which may reflect its severity.	(67)
TREM-1	Adults	STREM-1 has been explored as a potential diagnostic and severity biomarker for pneumonia.	(69)
TREM-1	Children	Measuring sTREM-1 may improve early triage, management, and prognosis in children with pneumonia at risk of mortality.	(70)
PSP	Adults	Plasma levels of Preepsin can be used for the diagnosis of pneumonia and predict ICU mortality in patients.	(71)
PSP	Adults	Elevated PSP levels indicate poor prognosis in hospitalized pneumonia patients and are associated with mortality.	(72)
PSP	Adults	The plasma levels of PSP upon admission may assess the severity of pneumonia in patients and predict 30-day mortality.	(73)

ProADM, pro-adrenomedullin; suPAR, soluble urokinase plasminogen activator receptor; TREM-1, triggering receptor expressed on myeloid cells 1; PSP, presepsin.

### Copeptin

Copeptin is a polypeptide secreted by the neuro-pituitary gland of the brain and is part of the C-terminus of the arginine vasopressin precursor. It has a long half-life and is easy to measure. Increased copeptin levels have been reported in adult patients with severe respiratory infections (63). Detection of copeptin levels may be of great significance for the early diagnosis, severity, and prognosis assessment of adult with severe pneumonia patients (64). A study has found that Copeptin appears to be a reliable and usable predictor for assessing the severity and prognosis of childhood pneumonia (65). Clinicians should evaluate the prognosis of children with severe pneumonia by timely detection of copeptin levels.

### Soluble urokinase plasminogen activator receptor (suPAR)

suPAR is a member of the plasminogen activation system, is distributed in plasma and body fluids, and reflects the body's immune and inflammatory levels. The concentration of suPAR in the plasma of healthy individuals is very low. During inflammation and infection, suPAR falls off the immune cell membrane, dissolves, and is released into the plasma, thereby activating LYMPHs and macrophages. It was found that the plasma suPAR levels in children with pneumonia were associated with severity (66, 67). It can be seen that early detection of plasma suPAR levels could reflect the severity of pneumonia in children.

### Triggering receptor expressed on myeloid cells 1 (TREM-1)

TREM-1 is a cell membrane receptor expressed on the surface of natural immune cells, NEUTs, and monocytes/macrophages. It exists in two forms: a soluble receptor (sTREM-1) and a membrane-binding receptor (mTREM-1). The former is not expressed in healthy people, but is increased in infectious diseases. It can inhibit the expression of anti-inflammatory mediators and promote the release of pro-inflammatory mediators (68). sTREM-1 is positively correlated with the severity of severe pneumonia in adult and pediatric patients (69, 70), and dynamic monitoring of sTREM-1 levels is of great significance for guiding doctors in clinical medication and evaluating the curative effect.

### Presepsin (PSP)

PSP, also known as sCD14-ST, is a soluble N-segment fragment secreted into plasma after CD14 cleavage. After bacterial infection, immune cells phagocytose bacteria, resulting in the increased secretion of PSP in the plasma, which is a sign that immune cells are activated. PSP is highly sensitive to bacterial infection. PSP, which is cleaved from the surface of monocytes and released into the bloodstream during the inflammatory response, is a newly identified biomarker for the early diagnosis and prognostic assessment of different types of infection and can distinguish the severity of infection. PSP is a biomarker for the early diagnosis of sepsis and its high levels are associated with higher mortality. Plasma PSP has good diagnostic and prognostic value for adult patients with pneumonia (71, 72). Plasma PSP levels have been shown to be a predictive biomarker for 30-day mortality in adult patients with severe pneumonia with high sensitivity and specificity (73). Few studies have examined the correlation between PSP and the prognosis of severe pneumonia in children. Large-scale clinical trials should be conducted to evaluate the efficacy of PSP before its application in routine clinical detection.

## Conclusions

Biomarkers related to the severity and prognostic value of severe pneumonia in children have shown good predictive value, which is helpful for clinicians in accurately judging the disease and implementing timely treatment. However, a single indicator is susceptible to multiple factors, causing poor repeatability and low specificity. The physiological fluctuation of CRP and PCT in infants is relatively large, and there is a lag in the prognosis evaluation of severe pneumonia. CRP has good diagnostic value for local bacterial infections. PCT is significant in systemic or severe infections, and it increases faster than CRP. PCT has important implications for guiding antibiotic therapy. NLR and PLR effectively integrate the three parameters of NEUT, LYMPH, and platelets to more accurately reflect the inflammatory status and avoid the fluctuation of a single index affecting the results. NLR reflects the immune and inflammatory status, whereas PLR indicates the level of systemic inflammation. The greatest advantage of IL-6 is its ability to respond promptly after infection. When diagnosing bacterial infections, the elevation of SAA is higher and the rate of decline during the recovery period of the disease is the fastest. In terms of viral infection, SAA level is significantly elevated, and CRP and PCT levels are slightly elevated. Therefore, the SAA is a sensitive indicator for the diagnosis of bacterial and viral infections. DD has an important guiding significance in determining the severity of infection, but there is currently no clear reference range to support this conclusion. PA and LDH are good predictors of myocardial damage in patients with severe pneumonia.

Most biomarkers have limitations in their applications. Currently, no biomarker has been found to have high sensitivity and specificity for the diagnosis of diseases. The combined detection of multiple biomarkers can improve judgment accuracy and increase clinical significance. Data on these common indicators mainly focus on admission detection and cannot reflect the condition after treatment. To better identify the degree of disease progression, it is necessary to dynamically monitor the changes in these indicators during treatment, more accurately evaluate the degree of disease progression, and carry out prognostic risk assessment. Given the current research status, we believe that dynamic detection of CRP, PCT, blood routine, IL-6, and SAA in high-risk children is helpful for assessing the severity of pneumonia. By detecting PA and LDH, we could better understand whether there is myocardial damage in children with pneumonia. We must emphasize that biomarkers may be useful, but they could not in any way replace the standard clinical approach. Doctors should be based on the patient's clinical manifestations, clinical scoring system, and comprehensive indicators combined with the rate of change analysis. The existing biomarkers have a certain value in evaluating the condition and prognosis of severe pneumonia in children, but there are still controversies in clinical practice. In the future, more studies are needed to further validate these findings, design more accurate assessment methods, and establish more accurate individualized treatment to maximize the ability to assess the severity and prognosis of severe pneumonia in children.
